# BDNF exon IV promoter methylation and antidepressant action: a complex interplay

**DOI:** 10.1186/s13148-022-01415-3

**Published:** 2022-12-26

**Authors:** Hansi Pathak, Anton Borchert, Sara Garaali, Alexandra Burkert, Helge Frieling

**Affiliations:** Laboratory for Molecular Neuroscience, Department of Psychiatry, Social Psychiatry and Psychotherapy, Hannover Medical School (MHH), 30625 Hannover, Germany

**Keywords:** BDNF exon IV promoter, Methylation, Antidepressants, Site-directed mutagenesis, MeCP2, CREB

## Abstract

**Background:**

BDNF exon IV promoter methylation is a potential biomarker for treatment response to antidepressants in MDD. We have previously shown CpG-87 methylation as a successful biomarker for the prediction of non-response to monoaminergic antidepressants like the SSRI Fluoxetine or the SNRI Venlafaxine. This study aimed to dissect the biological evidence and mechanisms for the functionality of CpG-87 methylation in a cell culture model.

**Results:**

We observed a significant interaction between methylation and antidepressant-mediated transcriptional activity in BDNF exon IV promoter. In addition, antidepressant treatment increased the promoter methylation in a concentration-dependent manner. Further single CpG methylation of -87 did not change the promoter activity, but methylation of CREB domain CpG-39 increased the transcriptional activity in an antidepressant-dependent manner. Interestingly, DNMT3a overexpression also increases the BDNF exon IV transcription and more so in Venlafaxine-treated cells.

**Conclusions:**

The study strengthens the previously reported association between antidepressant treatment and BDNF exon IV promoter methylation as well as hints toward the mechanism of action. We argue that potential CpG methylation biomarkers display a complex synergy with the molecular changes at the neighboring CpG positions, thus highlighting the importance of epiallele analyses.

**Supplementary Information:**

The online version contains supplementary material available at 10.1186/s13148-022-01415-3.

## Background

Major depressive disorder (MDD) is a multifaceted disease disorder that imposes a measurable burden on society. Over the past decade, it has been widely accepted that symptoms and manifestations of depression between individuals cannot be empirically explained by dysfunction of any particular biological process. Although matching the pathology to a certain extent with the molecules and pathways involved can draw a subjective classification, more evidence is needed to extrapolate findings to the therapeutic regimens [1]. The major challenge in treating the patients with MDD is the inherent heterogeneity in response rates toward the commonly used antidepressants, the serotonin reuptake inhibitors (SSRI) and serotonin–norepinephrine reuptake inhibitors (SNRI) [2].

Therefore, it is imperative to work systematically toward understanding the underlying causative and predictive biological differences in the patients.

Brain-derived neurotrophic factor (BDNF)-mediated signaling cascades are implicated in neuronal differentiation, synaptic plasticity and neurotransmission, claiming BDNF as a central molecule for various interventions for depression as well as other psychiatric disorders. Compelling evidence is available toward the disruption of BDNF gene in manifesting the depressive behaviors from both animal and patient studies [3]. BDNF upregulation is one of the key functional phenomena to downstream pathways of antidepressant drugs [4, 5].

Transcription of BDNF is an intricate example of temporal and spatial regulation. The BDNF gene contains 11 exons with multiple splicing sites [6]. The last exon codes for BDNF protein and contains two poly (A) stretches resulting in a protein with a short or long 3′ UTR. The remaining 10 exons contain independent internal promoters that allow the transcription of 13 different transcripts each containing a unique 5′ UTR proximate to the coding exon [7]. Transcription factors, epigenetic modifiers and RNA-binding proteins work in synchrony to regulate, modulate and fine-tune the expression of specific BDNF transcript in response to developmental cues and also various environmental stimuli [8].

Neuronal activity-dependent calcium influx is important to process and store the information in response to environmental stimuli. BDNF exon I and IV transcription is regulated by calcium-dependent transcription factors, cAMP response element-binding protein (CREB) and upstream stimulating factor (USF) [9]. CREB is a histone deacetylase and recruits other transcriptional activators, upregulating the transcription of BDNF transcripts [10]. In addition, histone modification like the H3K4me3 mark has been associated with active BDNF promoter [11, 12].

Interestingly, transcriptional regulation of BDNF, in absence of any stimuli occurs via CpG methylation or H3K27 trimethylation. Methylation-dependent regulation has been reported for BDNF exon I, IV, VI and IX [13–15].

MDD has a mere heredity of 37%, and therefore the onset, development and progression of disease have been shown to be primarily mediated via epigenetic mechanisms [16]. Social factors like early life stress, childhood maltreatment and maternal separation change BDNF promoter methylation [17]. Serum BDNF levels before treatment have been associated with SSRI response in depression [18]. CpG-specific hyper- and hypomethylation of exon I and IV promoters have been reported in various psychiatric disorders including MDD. Although it remains inconclusive whether antidepressants alter BDNF methylation, differences in the CpG methylation of BDNF internal promoters have been presented as a biomarker for therapy response [19, 20].

We have previously shown that the hypermethylation at CpG-87 (from the transcriptional start site) can successfully predict the non-response to antidepressant therapy [21]. This finding has been replicated in a larger cohort of moderate to severely depressed patients, while it could not be replicated for the patients with mild or minor depression [22]. Similar findings have been published in a Chinese cohort [23]. In our first report, we also provided first in vitro data on the transcriptional activity of the BDNF exon IV promoter. Antidepressant incubation with Fluoxetine (SSRI) or Venlafaxine (SNRI) led to reduced activity in a dual-luciferase reporter assay. This down-regulation was specific to unmethylated promoter fragment.

**Fig. 1:**
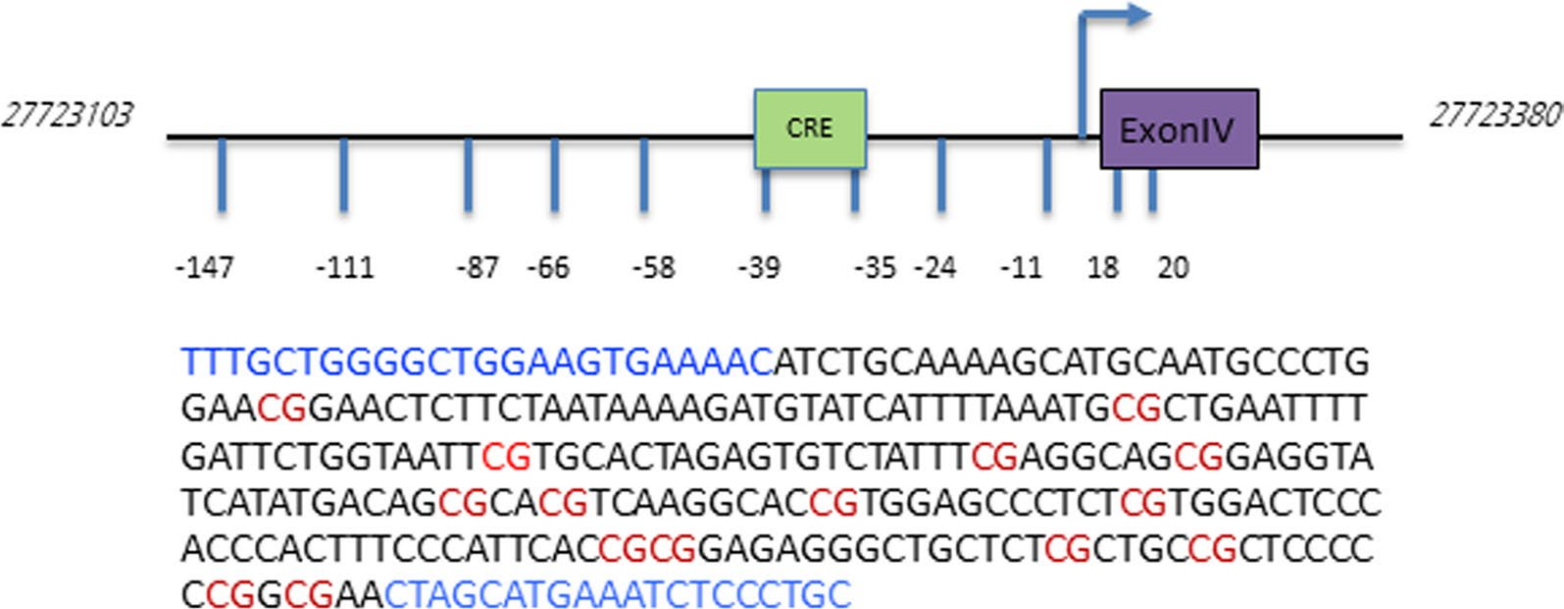
BDNF exon IV promoter schematics. Sequence of the BDNF exon IV promoter analyzed for methylation. Primers (blue) and the CpGs (red) are highlighted. CpG-39 and -35 encompasses the cAMP response element (CRE). CpGs are numbered with respect to BDNF exon IV promoter

Here, we aim to provide a more detailed insight into the complex interplay of single CpG methylation and antidepressant effects on the BDNF exon IV promoter activity and BDNF expression (Fig. 1).

## Results

### Transcriptional activity of BDNF exon IV correlates with promoter methylation

**Fig. 2:**
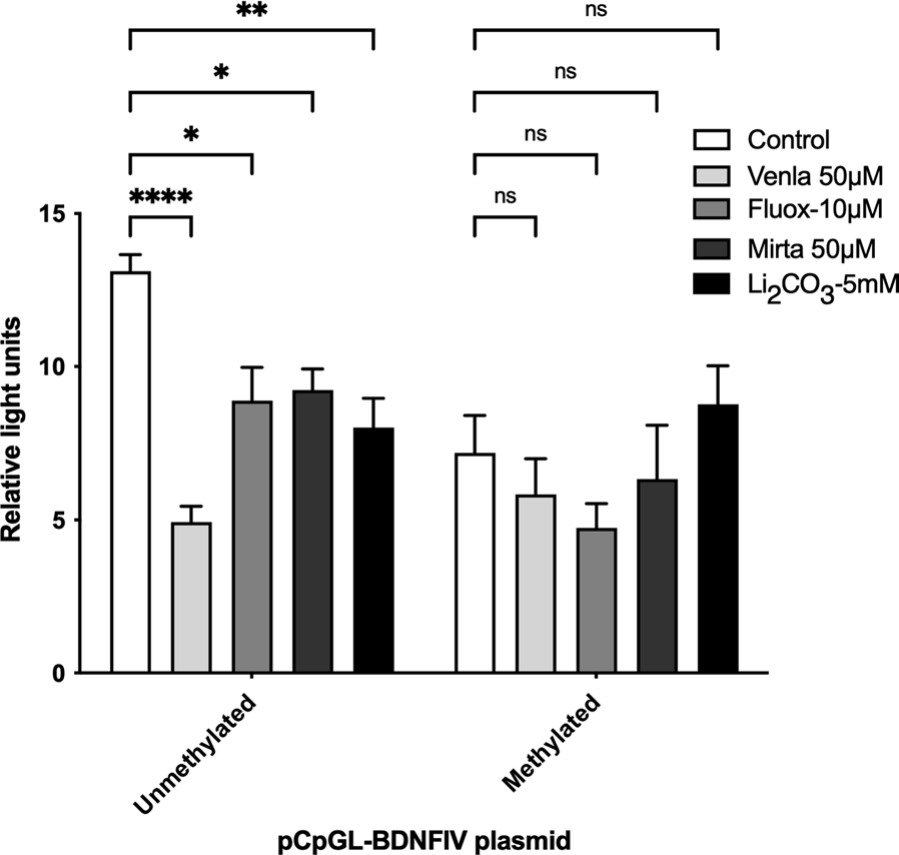
**a** Effect of antidepressants on BDNF exon IV promoter activity. The antidepressants at the given concentration decrease the promoter activity: Venlafaxine (50 µM, *P* < 0.001), Fluoxetine (10 µM, *P* < 0.05), Mirtazapine (50 µM, *P* < 0.05) and Lithium carbonate (3 mM, *P* < 0.01). Overall, methylation reduces the promoter activity (ANOVA, main effect of methylation: *P* = 0.0008). There is no significant reduction in promoter activity on AD treatment in methylated fragment

We cloned BDNF exon IV promoter region in a CpG-free vector upstream of the luciferase gene and analyzed the effect of antidepressants on the transcriptional activity. This experiment replicates the findings from our previous paper [21]. We observed a decrease in the BDNF exon IV transcription of unmethylated promoter (*F*_(1, 50)_ = 11.35; *P* = 0.0015) in SH-SY5Y neuroblastoma cell line after treatment with antidepressants. The observed reduction was significant for Venlafaxine, Lithium carbonate, Fluoxetine, and Mirtazapine (*P* = 0.05). Interestingly, the antidepressant-induced reduction in BDNF exon IV transcription was not seen in methylated promoter, thereby explaining a significant interaction between methylation and antidepressant on the promoter activity (*F*_(4, 50)_ = 4.059; *P* = 0.0063). An overall reduction in promoter activity was observed in methylated promoter construct (main effect derived from the two-way ANOVA (*F*_(4, 50)_ = 5.615; *P* = 0.0008)) (Fig. 2). In the post hoc analysis, we observed a strong and significant reduction in activity in the methylated promoter fragment without antidepressant incubation (control) as compared to the unmethylated construct (post hoc: t_(50)_ = 3.948; *P* < 0.01).

### Effect of single CpG methylation on BDNF exon IV transcriptional regulation

Clinical data from our previous studies highlighted the importance of CpG-87 methylation for the efficacy of ADs. Patients with methylated CpG-87 showed an increase in BDNF plasma levels after 14 days of treatment. To model the clinical effect of CpG-87, we mutated the cytosines (-87, -39 and -35) to methylated cytosines in BDNFIV-pCpGL construct and compared it to control construct. Additionally, we incubated transfected cells with Venlafaxine and Fluoxetine at the given concentrations for 48 h and harvested cells for luciferase assay. We observed a significant overall effect of methylation (*F*_(3, 151)_ = 49.41; *P* < 0.0001). No significant effect of ADs was observed (*F*_(4, 151)_ = 2.23; *P* = 0.06).

**Fig. 3:**
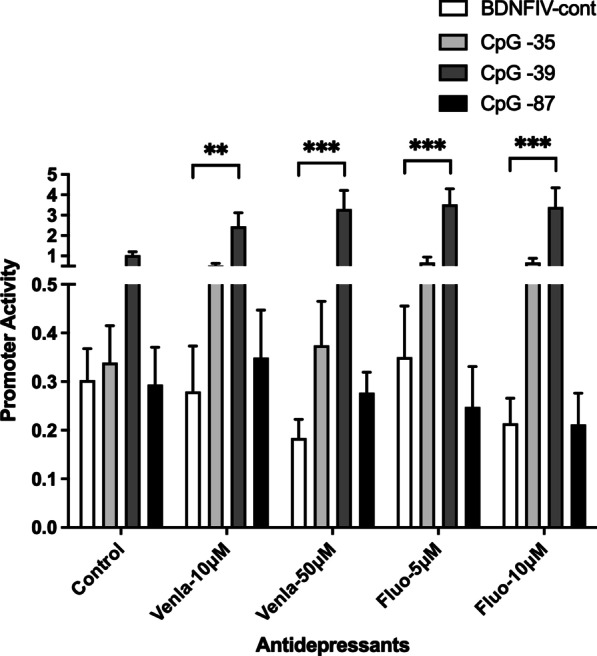
Effect of single CpG methylation on BDNF exon IV promoter activity. Methylation at CpG position -35, -87 and -39 does not show any significant change in promoter activity in control condition. In the cells treated with ADs, CpG-39 methylation significantly increases the promoter activity (*P* < 0.0001). Methylation at CpG-35 and -87 had no significant effect

We observed an interaction between CpG methylation and AD treatment (*F*_(12, 151)_ = 1.86; *P* = 0.043). This effect was mainly driven by CpG-39 methylation, as the methylated construct showed significant differences in promoter activity in comparison with BDNFIV unmethylated control only upon AD treatment (Venlafaxine 10 µM, *P* < 0.01; Venlafaxine 50 µM, Fluoxetine 5 µM and 10 µM, *P* < 0.0001) (Fig. 3).

### Effect of antidepressants on BDNF exon IV promoter methylation levels

To test the effect of Venlafaxine and Fluoxetine on BDNF exon IV promoter methylation in a drug-naïve environment, we incubated SHSY-5Y cells at two different concentrations (Venlafaxine 10 µM and 50 µM; Fluoxetine 5 µM and 10 µM) of drugs and harvested cells at four different time points (0.5 h, 2 h, 24 h and 48 h).

**Fig. 4:**
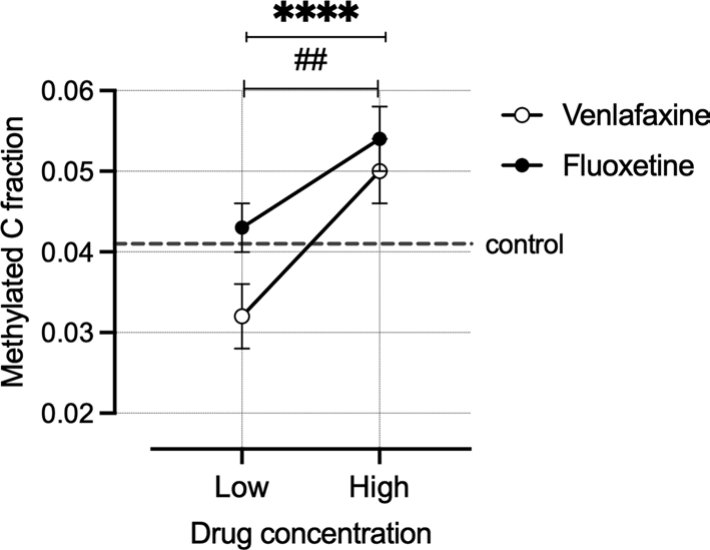
Antidepressants increase BDNF exon IV promoter methylation. Venlafaxine at 50 µM significantly increases BDNF exon IV promoter methylation in comparison with 10 µM (^*^P < 0.0001) and 10 µM Fluoxetine in comparison with 5 µM (^#^*P* = 0.025)

We used a linear mixed model computing methylation as a dependent variable and CpG position, drugs, concentration and time point as factors. We observed a significant effect of CpG position (*F*_(10, 1301)_ = 69.482; *P* < 0.0001). A significant effect of drugs was found on the BDNFIV methylation (*F*_(1, 1301)_ = 5.44; *P* = 0.02). Venlafaxine mainly drove this effect (Fig. 4). The drug concentration had a significant effect on the methylation for both Venlafaxine (*F*_(1, 241)_ = 13.90; *P* < 0.0001) and Fluoxetine (*F*_(1, 276)_ = 5.706; *P* = 0.025). No overall effect of time point was observed, but a significant change in methylation occurred over time in Fluoxetine-treated cells (*F*_(3, 276)_ = 2.789; *P* = 0.041) (data not shown).

### Effect of DNMT overexpression on BDNF exon IV methylation and transcriptional regulation

To get an insight into methylation and BDNF exon IV transcription, we co-transfected the luciferase construct harboring the BDNF promoter with DNMT3a, DNMT3b and DNMT1 mammalian expression vectors and their empty vector controls pHTC (DNMT1) and pCDNA3 (DNMT3a and 3b). We found that DNMT3a increases the BDNF exon IV promoter activity whereas DNMT3b and DNMT1 have no effect.

**Fig. 5:**
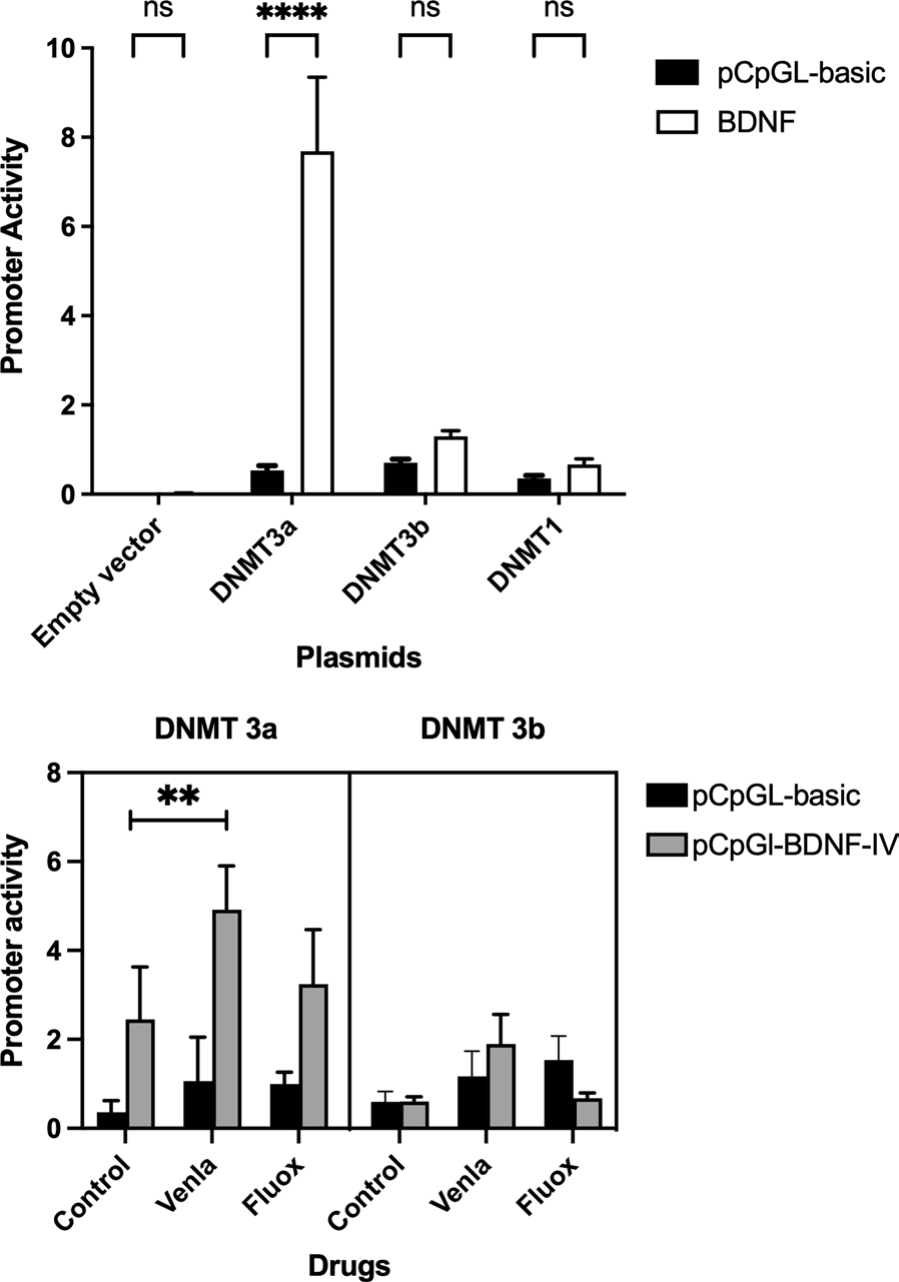
Effect of DNMT overexpression on BDNF exon IV promoter activity. **a** DNMT 3a overexpression significantly increases BDNF exon IV promoter activity (*P* < 0.0001). DNMT3b and DNMT1 do not have any significant effect. **b** Venlafaxine further increases the DNMT3a-mediated increase in promoter activity (*P* < 0.01). Fluoxetine has no significant effect

Further, we treated the transfected cells with ADs to investigate, if overexpression of methyltransferases has any effect on BDNF transcriptional activity in the presence of ADs, we observed a significant effect of the drugs (*F*_(2, 24)_ = 9.33; *P* = 0.001). The DNMT3a-mediated increase on promoter activity was replicable (*F*_(3, 24)_ = 29.03; *P* < 0.0001) (Fig. 5a, b).

Additionally, we sequenced the cells transfected with DNMTs for methylation analysis to address, if DNMT overexpression has a direct effect on BDNF exon IV promoter CpG methylation. We also used nanaomycin as a chemical modulator as it has been shown to inhibit DNMT3b. We observed no significant effect of DNMT overexpression or nanaomycin treatment on BDNF exon IV promoter methylation.

## Discussion

BDNF exon IV promoter activity is regulated by methylation in a CpG-dependent manner in the absence of neuronal activity [8]. BDNF exon IV promoter methylation has been associated with multiple psychiatric disorders along with MDD [19] as well as treatment response to ADs [24] and other available therapy regimens [25]. Previous work from our lab has confirmed the CpG-87 hypomethylation as a biomarker for unsuccessful AD treatment in two different studies [21, 22]. This work aimed to explore the link between AD action and CpG-87 methylation mechanistically.

To understand the effect of ADs, methylation and their interaction on the transcriptional activity of BDNF exon IV, we cloned BDNF exon IV promoter region in a CpG-free luciferase vector [26] and transfected it into a neuroblastoma cell line SH-SY5Y and treated them with ADs 24 h after transfection for 48 h. The ADs decreased BDNF exon IV promoter activity and this effect was nullified by methylation arguing in favor of a possible link between antidepressant-mediated BDNF increase in serum of patients harboring methylation at CpG-87 locus. These findings corroborated with our previous study where we used the luciferase vector (Promega) containing CpGs demonstrating that the reduction in the promoter activity is due to the CpG methylation of BDNF exon IV promoter and not the vector itself. It must be noted that we methylated the promoter fragment using bacterial methylase M.SssI, thereby methylating every CpG in the promoter to approximately 90% that does not correspond to the physiological methylation pattern.

The linear effect of pharmacological treatments on BDNF exon IV promoter methylation has not been clearly elucidated, mainly because of the lack of pre-treatment methylation data from the patients and lack of drug-naïve cohorts [19]. The clinical data from our previous study show that antidepressants do not change the methylation over 6 weeks of treatment [21]. This patient cohort did not contain any drug-naïve patients, and therefore we could have missed potential AD-mediated changes in methylation. To address this in vitro, we incubated SH-SY5Y cells with ADs and analyzed the CpG methylation of BDNF exon IV promoter. Both Venlafaxine and Fluoxetine increased the mean promoter methylation in a concentration-dependent manner. We did not observe any change in the methylation of CpG-87 upon treatment, suggesting that the treatment itself does not directly modulate CpG-87. CpG-147, CpG-66, CpG-39 and CpG-35 (Additional file 1: Fig. S1) drove the differences in the mean methylation. Our data justify the scope for further studies investigating the molecular mechanisms underlying the antidepressant moderated epigenetic changes.

Further, to differentiate the regulatory effect of methylation of individual CpGs from the whole promoter, we mutated the CpGs of interest to methylated CpGs in the BDNFIV-pCpGL basic construct and performed luciferase assays with or without ADs. We included CpG-87 to model the clinical findings and CpG-35 and -39 due to their proximity with CRE (CREB binding element). CREB has been shown to regulate BDNF protein [27–29] and is involved in antidepressant response [30]. We observed no difference in the BDNF exon IV promoter activity upon methylating CpG-35, and -87 in comparison with the unmethylated controls, but CpG-39 methylation increased the transcriptional activity of the promoter. Further plasmid harboring methylated CpG-39 showed a significant increase in promoter activity in the Venlafaxine- and Fluoxetine-treated cells, suggesting yet again possible drug–methylation interaction.

**Fig. 6:**
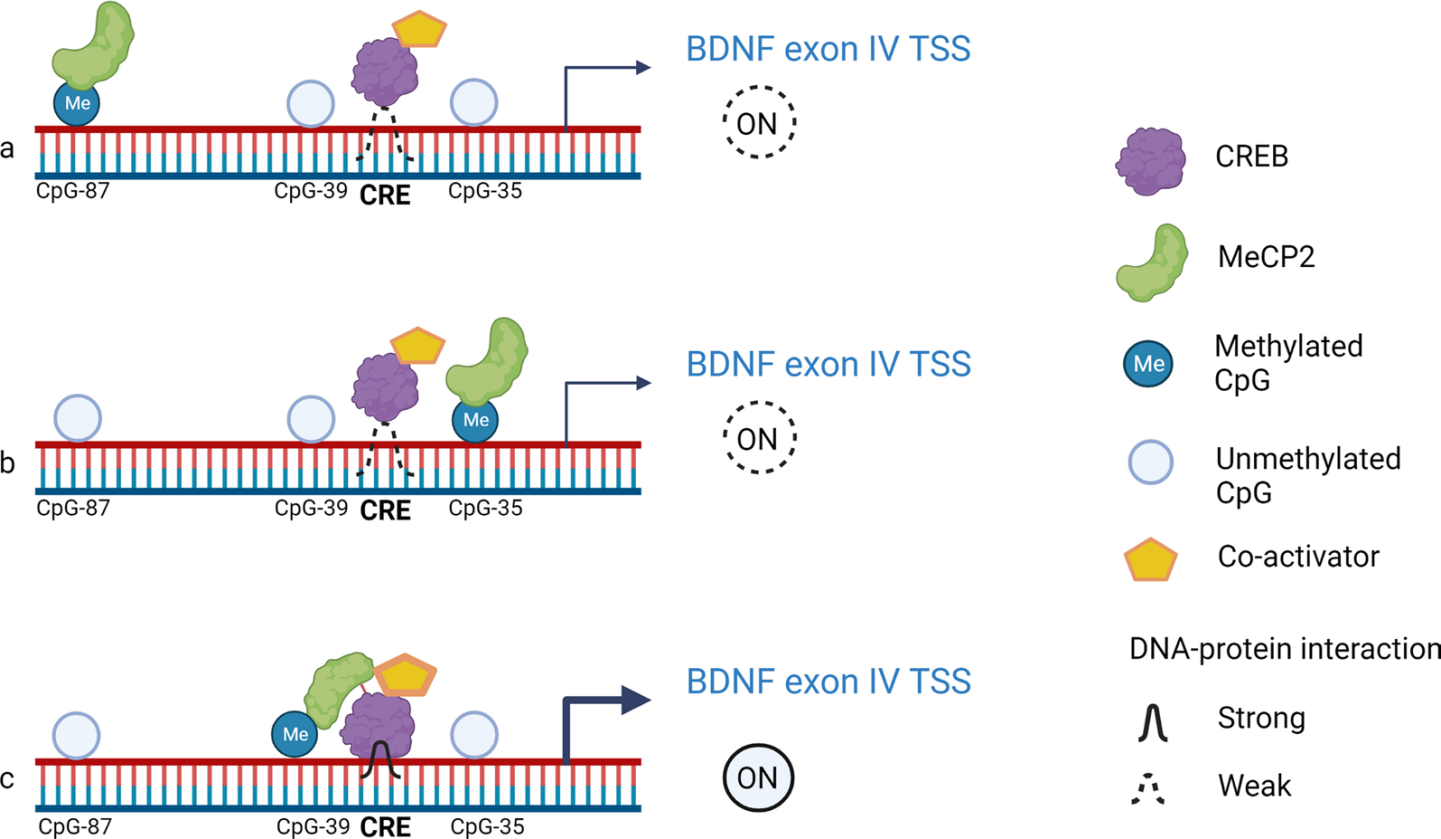
Proposed molecular mechanism for methylation-specific BDNF exon IV transcriptional activation. CREB, a known transcriptional factor binds to cAMP response element (CRE) on BDNF exon IV promoter and upregulate transcription by recruiting co-activators like CREB-binding protein (CBP). MeCP2 binds across genome, preferably to methylated CpGs and functions as chromatin modifier. Site-specific methylation of BDNF exon IV promoter increases MeCP2 occupancy at the methylated CpGs. MeCP2 and CREB form a transcriptional activating complex. No increase in CREB–CRE interaction was observed, if methylated CpG is far from CRE as with CpG-87 (**a**) or at the 3′ end of CRE as observed with CpG-35 (**b**). Methylation at CpG-39 allows the recruitment of MeCP2-CREB protein complex and increases the transcriptional activity at BDNF exon IV promoter (**c**). Illustration was created with BioRender.com

Methyl-CpG-binding protein (MeCP2), an important epigenetic regulator, [31] has been implicated in depression and other psychiatric disorders [32]. MeCP2 can bind to both methylated cytosine (5mC) and 5-hydroxymethylcytosine (5hmC), thereby acting as both transcriptional repressor and activator [33]. Studies have shown that MeCP2 binds to methylated CpGs on BDNF exon IV promoter and represses transcription by blocking CRE [8] or recruiting CREB to a repressor complex [34]. Contradictorily, a strong line of evidence suggests MeCP2 physically and functionally interacts with CREB facilitating the interaction with CRE on the BDNF and other active promoters [35]. Therefore, we speculate that the observed increase in transcriptional activity can be due to enhanced MECP2-CREB complex recruitment to the promoter (Fig. 6).

Antidepressants have been shown to phosphorylate MeCP2 at S421 [36], but we did not observe MeCP2 phosphorylation in SH-SY5Y cells upon antidepressant treatment after 48 h (data not shown). Furthermore, no clear evidence exists for dissociation of MeCP2 from DNA upon calcium influx-induced neuronal depolarization and phosphorylation at specific serine 421 [37], which has been speculated to be a potential mechanism of antidepressant action [38]. Compelling evidence arguing against an important role of S421 phosphorylation for the DNA binding capacity of MeCP2 has been reported by Cohen and co-workers. In S421A mice lacking a functional serine phosphorylation site, no significant change in MeCP2-DNA binding profile was observed in cortical neurons [39].

Therefore, we speculate that the antidepressant-mediated increase in BDNF exon IV promoter activity in the methylated CpG-39 promoter construct is due to an increase in promoter methylation at the CpGs that synchronously upregulate the transcription.

We also conclude that CpG-87 methylation does not directly increase BDNF exon IV transcription. A unique population of cells might contribute to the association of positive treatment response to the methylation (1–5%) at this position (measured in blood), which can be identified by emerging techniques like single-cell epigenomics [40]. Due to technical limitation, we could only methylate single CpG at a time, so we did not account for permutation and combination of methylation patterns that probably exist in the patients. We will be sequencing a larger cohort of MDD patients using Oxford Nanopore Technologies that allows single-strand methylation analysis that can dissect complex epi-allelic methylation patterns.

DNA methyltransferases specifically methylate regulatory CpGs and modulate gene transcription. A suggestive link exists between DNMTs expression and MDD in the literature [41]. To address whether the AD-dependent BDNF increase in serum/plasma levels of patients and animals is associated with in vitro methylation changes, we overexpressed DNMTs and treated the cells with ADs. Of the methyltransferases, only DNMT3a increased exon IV activity in control and Venlafaxine-treated cells. Interestingly, of the two de novo methyltransferases, DNMT3a is expressed in adult brain, whereas DNMT3b is only found in neural progenitor cells [17]. When we bisulfite-sequenced the DNMT overexpressed cells, we did not observe any change in the CpG methylation at BDNF exon IV promoter. Taken together, our findings suggest an indirect effect of DNMT3a overexpression on an increase in BDNF exon IV promoter activity. It will be very interesting to get a deeper insight into the expression pattern of DNMTs in the responders and non-responders.

Our system relies on artificially curated methylation marks that do not reflect the complex scenario in vivo. Therefore, it will be interesting to use Cas9-mediated methylation editing by recruiting methyl modifiers (DNMT/TET) to study the interaction between BDNF exon IV promoter methylation and antidepressant action on its regulation. Another limitation of the study is that we used a neuroblastoma cell line, thus lacking the communication between different cell types that is essential for antidepressant action. Therefore, we plan to replicate the findings in a mixed cell culture model consisting neurons, astrocytes and myelinating oligodendrocytes.

## Conclusions

Our study provides evidence for AD-dependent methylation changes on the BDNF exon IV promoter and emphasizes the importance of studying such interactions in the drug-naïve cohorts of MDD patients. It also describes the functional relevance of studying CpG-specific methylation and its cross talk with AD-mediated increase in BDNF exon IV promoter activity. Further, we highlight the importance of studying DNMTs, especially DNMT3a in the context of MDD.

## Methods

### Cell culture

Human neuroblastoma cell line SH-SY5Y (ATCC, Wesel) was cultured in DMEM-Ham’s F12 medium 1:1 (Biochrom AG, Berlin) supplemented with 10% FBS Superior (Biochrom AG, Berlin) and 1% penicillin/streptomycin (PAN Biotech GmbH, Aidenbach). Cells were maintained at 37 °C in a humidified atmosphere of 5% CO_2_ in atmosphere. Cells were incubated with indicated concentration and for the indicated time with the drugs.

### Insert amplification and cloning

BDNF exon IV promoter region (GRCh38; chr11: 27701325-27702402) was amplified (primers: Inf-BDNF-pCpGL Fw/Rv) from the genomic DNA using KOD Hot Start Taq DNA Polymerase (Novagen, Merck Chemicals, Darmstadt, Germany). Primers contained pCpGL CpG-free luciferase vector-specific overhangs (red) that were adapted to the PCR product. The 1172 bp fragment with adapter sequences was cloned into HindIII HF-linearized pCpGL firefly luciferase reporter plasmid [26] by recombination using In-Fusion® HD Cloning Kit (Clontech, cat. no. 639648) (Additional file 2: Fig. S2.). Similarly, DNA methyltransferase 1 (DNMT1) cDNA were PCR amplified (primers: Inf-DNMT1-halo Fw and Rv) from a mixture of human cell line cDNA using KOD Hot Start Taq DNA Polymerase and cloned in pHTC HaloTag®CMV-neo Vector (Promega, cat. no. G7711). The plasmids pcDNA3/Myc-DNMT3a and pcDNA3/Myc-DNMT3b were gifts from Arthur Riggs (Addgene, cat. no. 35521 and cat. no. 35522, respectively [42]. Plasmids were transformed in NEB alpha competent cells except pCpGLempty and pCpGL-BDNFIV that were transformed in piR1 Competent E. coli (Invitrogen) with 5 µL recombined plasmid and plated on LB-Agar containing 100 µg/mL Ampicillin: Grown colonies were transferred to 3 mL LB-Amp-Medium and plasmids were isolated using the NucleoSpin Plasmid Kit (Macherey–Nagel, Düren). After verifying the plasmids containing the hBDNF exon IV promoter region by sequencing (primer: pCpGL-seq_Fw), DNA purification was done using endotoxine-free plasmid isolation kit (NucleoBond Xtra Midi EF Kit; Macherey–Nagel, Düren). Expression of DNMT1, DNMT3a and DNMT3b was validated by western blotting (data not shown). Refer to Table 1 for primer sequences. The underlined sequence is gene-specific.

**Table 1:** Primer list

Name	Sequence (5′-3′)
Inf-BDNF-pCpGL Fw	GTGGATCCAGATCTT**ATTCCTCTGATACCCAG**
Inf-BDNF-pCpGL Rv	CTCCATGGACTAAGCT**CCACCTTTTCAGTCAC**
pCpGL-seq_Fw	AAAGGAATTCCTGCAGGACTAGTG
pCpGL-seq_Rv	TTGGCATCCTCCATGGACTAAGCT
bisBDNF_IV_forw1:	GGGGGAGGATTAATTGAGTTAGTTTTG
bisBDNF_IV_forw2:	TTTGTTGGGGTTGGAAGTGAAAAT
bisBDNF_IV_rev:	ATA TAT ACT CCT TCT ATT CTA CAACAA
BDNF_IV_seq:	ACAAAAAAATTTCATACTAA
Inf-DNMT1-Halo-Fw	TCACTATAGGGCTAGC**AGATGCCGGCGCGTACCGCC**
Inf-DNMT1-Halo-Rv	TCCGCGGTAGGAATTC**GTCCTTAGCAGCTTCCTCCTCCTTTA**
CpG87-mut-Fw	TCTGGTAATTmCGTGCACTAGAG
CpG87-mut-Rv	ATCAAAATTCAGCGCATTTAAAATG
CpG35_NEB-mut-Fw	TGACAGCGCAmCGTCAAGGCAC
CpG35_NEB-mut-Rv	TATGATACCTCCGCTGCC
CpG39_NEB-mut-Fw	CATATGACAGmCGCACGTCAAGGC
CpG-39_NEB-mut-Rv	ATACCTCCGCTGCCTCGA
CpG87-NEB-Control-Fw	TCTGGTAATTCGTGCACTAGAG
CpG87-NEB-Control-Rv	ATCAAAATTCAGCGCATTTAAAATG

Gene-specific sequence is highlighted in the primers used for cloning

### In vitro methylation of BDNF exon IV promoter construct

The pCpGL-BDNF IV promoter reporter (4 µg) was incubated (4 h at 37 °C) with CpG Methyltransferase M.SssI (20 U; New England Biolabs, Ipswich, MA) in buffer containing 640 µM S-adenosylmethionine (New England Biolabs, Ipswich, MA). Subsequently, the methylated plasmid was purified with NucleoSpin Plasmid Kit (Macherey–Nagel, Düren).

### Site-directed mutagenesis

CpGs-87, -39 and -35 were methylated using the Q5 Site-Directed Mutagenesis Kit (NEB, E0554) following the manufacturer’s protocol. Briefly, the pCpGL-BDNFIV promoter reporter construct was reverse-amplified using mut -87, mut -39 and mut -35 or mut-cntrl Fw/Rv primers and Q5 Hot Start High-Fidelity DNA Polymerase. The primers were back-to-back primers and were designed using NEBaseChanger. Cytosine at the desired position was replaced with methylated cytosine. Multiple reactions were pooled to gain a sufficient starting DNA amount. The PCR product was combined with the KLD Mix, containing a blend of kinase, ligase and DpnI enzymes, and was incubated for 15 min. After KLD treatment, the reaction was purified using NucleoSpin Plasmid Kit (Macherey–Nagel) and was used directly for transfection. The efficiency of mutagenesis was assessed by Sanger sequencing. All three CpGs were about 60% methylated. Refer to Table 1 for primer sequences.

### Transfection and luciferase-based reporter gene assays

1.5 × 10^5^ SH-SY5Y cells were plated in 96 well plates for 24 h before transfection. 100 ng/well of pCpGL-BDNFIV promoter luciferase reporter plasmid or methylated pCpGL-BDNFIV promoter luciferase reporter plasmid were co-transfected with 2 ng/well pGL4.74 [hRluc/TK] renilla luciferase control plasmid (Promega, Madison, WI) for normalization (3 columns per reporter plasmid) using Lipofectamine 3000 (company). 50 ng of the luciferase constructs was used for transfection, both for control and for mutated CpGs. Cells were harvested 48 h after transfection. For DNMT overexpression experiments, 25 ng of DNA was used for transfection for both DNMTs and empty vectors. Antidepressants were added 12 h post-transfection. After incubation over 48 h, cells were lysed and luminescence was measured using the Dual-Luciferase Reporter Assay System (Promega, Madison) and the GloMax-Multi + Luminometer (Promega, Madison), according to the manufacturer’s protocol.

### Luciferase assays

Post-transfection, media was removed and cells were washed with PBS. Assay was performed using Dual-Luciferase Reporter Assay System (Promega), and the luminescence was recorded using a double injector program on GloMax-Multi Detection System. The assay was applied in triplicates and was repeated 3 times. For analysis, firefly readings were normalized by the respective renilla luminescence readings and normalized values were imported to GraphPad Prism 5 (Graph Pad Inc., San Diego, CA).

### Bisulfite sequencing of the BDNF exon IV promoter region

Genomic DNA was extracted from cells using NucleoMag Blood 200µL (Macherey–Nagel) according to the manufacturer’s protocol. Afterward, 500 ng of genomic DNA was modified by sodium bisulfite using the EpiTect® 96 Bisulfite Kit (QIAGEN).

Primers were designed to amplify a region covering a fragment of 277 base pairs (bp) from − 200 to + 77 bp relative to the starting bp of the BDNF exon IV promoter (NCBI NC_000011.9: 27723103-27723380) containing 13 CpG sites within the promoter region of BDNF exon IV. To amplify a specific product, a semi-nested polymerase chain reaction (PCR) was performed using 1 µL bisulfite modified DNA or PCR round 1 product with 0.4 µL of each primer and 5 µL HotStarTaq Master Mix (Qiagen) filled to a reaction volume of 10 µL.

Sequencing of the reverse strand was performed using a BigDye® Terminator v3.1 Cycle Sequencing Kit (Applied Biosystems, Foster City, CA, USA) on an Applied Biosystems® 3500xL Genetic Analyzer (Applied Biosystems; POP-7™ polymer) according to the manufacturer’s instructions. Primer sequences are given in Table 1 (Amplification: bisBDNF_IV_forw1/2 and bisBDNF_IV_rev; Sequencing: BDNF_IV_seq). Refer to Table 1 for primer sequences.

### Statistical analysis

The methylation of each of the 11 CpGs in the CpG island was assessed using the Epigenetic Sequencing Methylation analysis Software (ESME) [43] which compares each methylation site to the original sequence of the promoter. To analyze the effect of antidepressant treatment on BDNF exon IV promoter methylation, mixed linear models for repeated measurements were performed including the factors: CpG position, drugs (Venlafaxine/ Fluoxetine), time point (0.5 h, 2 h, 24 h and 48 h) and drug concentration (5 µM and 10 µM for Fluoxetine and 10 µM and 50 µM for Venlafaxine). For the model assessing the effect of DNMTs and nanaomycin, CpG position and methyl modulators were used as factors. Data were analyzed employing IBM SPSS Statistics for Windows, Version 21.0. (Armonk, NY: IBM Corp.) and illustrated using Graph Pad Prism 5 (Graph Pad Inc., San Diego, CA). All the luciferase data were analyzed using two-way ANOVA in GraphPad Prism 5.

## Supplementary Information

**Additional file1**. **Figure S1**: Effect of antidepressants on single CpG methylation after 48 h of incubation with Venlafaxine (50 µM, 10 µM) and Fluoxetine (5 µM, 10 µM).

**Additional file2**. **Figure S2** Plasmid map of pCpGL-BDNFIV construct. BDNF exon IV promoter insert (orange) is cloned upstream of luciferase gene (neon green). Beginning of exon IV is indicated (grey). The PCR-amplified fragment used for methylation analysis (yellow) and the CpGs (pink) are marked. The transcription factor binding sites CARE1 and CRE are highlighted (purple). The plasmid map was made using Benchling [Biology Software]. (2022). Retrieved from https://benchling.com.

## Data Availability

The datasets used and/or analyzed during the current study are available from the corresponding author upon reasonable request.

## Abbreviations

AD: Antidepressants
BDNF: Brain-derived neurotrophic factor
CREB: CAMP response element-binding protein
CBP: CREB-binding protein
DNMT: DNA methyltransferase
MDD: Major depressive disorder
MeCP2: Methyl-CpG-binding protein 2
SSRI: Serotonin reuptake inhibitors
SNRI: Serotonin–norepinephrine reuptake inhibitors
