# Supplementary figures and images for: BDNF exon IV promoter methylation and antidepressant action: a complex interplay
Source: Clin Epigenetics. 2022 Dec 26;14:187. doi: 10.1186/s13148-022-01415-3 (PMC9793565; doi:10.1186/s13148-022-01415-3)

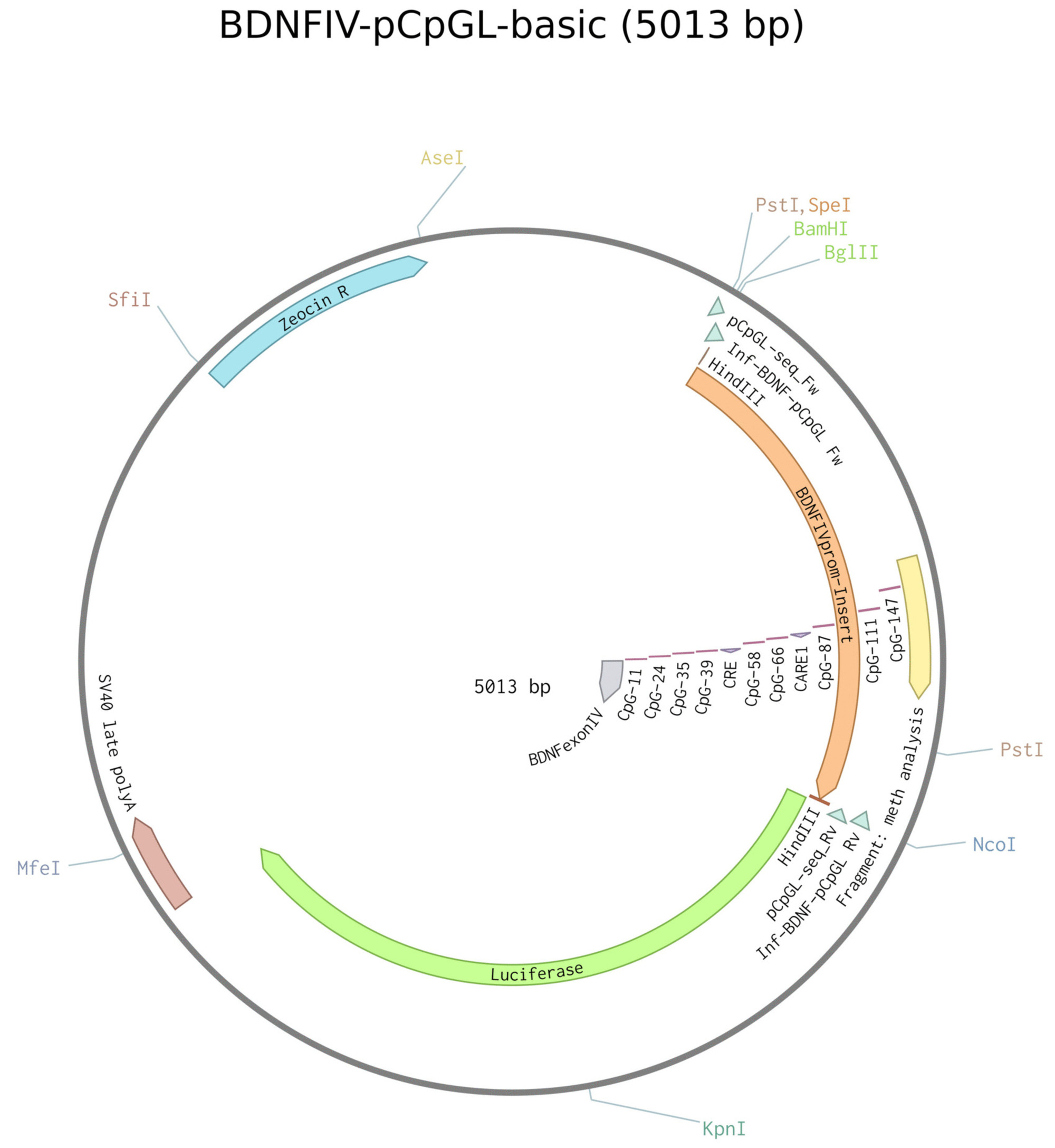

Supplement: Supplementary file 2 — Additional file2. Figure S2 Plasmid map of pCpGL-BDNFIV construct. BDNF exon IV promoter insert (orange) is cloned upstream of luciferase gene (neon green). Beginning of exon IV is indicated (grey). The PCR-amplified fragment used for methylation analysis (yellow) and the CpGs (pink) are marked. The transcription factor binding sites CARE1 and CRE are highlighted (purple). The plasmid map was made using Benchling [Biology Software]. (2022). Retrieved from https://benchling.com. [file 13148_2022_1415_MOESM2_ESM.tif]
