# Supplementary material for: BDNF exon IV promoter methylation and antidepressant action: a complex interplay
Source: Clin Epigenetics. 2022 Dec 26;14:187. doi: 10.1186/s13148-022-01415-3 (PMC9793565; doi:10.1186/s13148-022-01415-3)
Supplement: Supplementary file 2 [file 13148_2022_1415_MOESM1_ESM.docx]

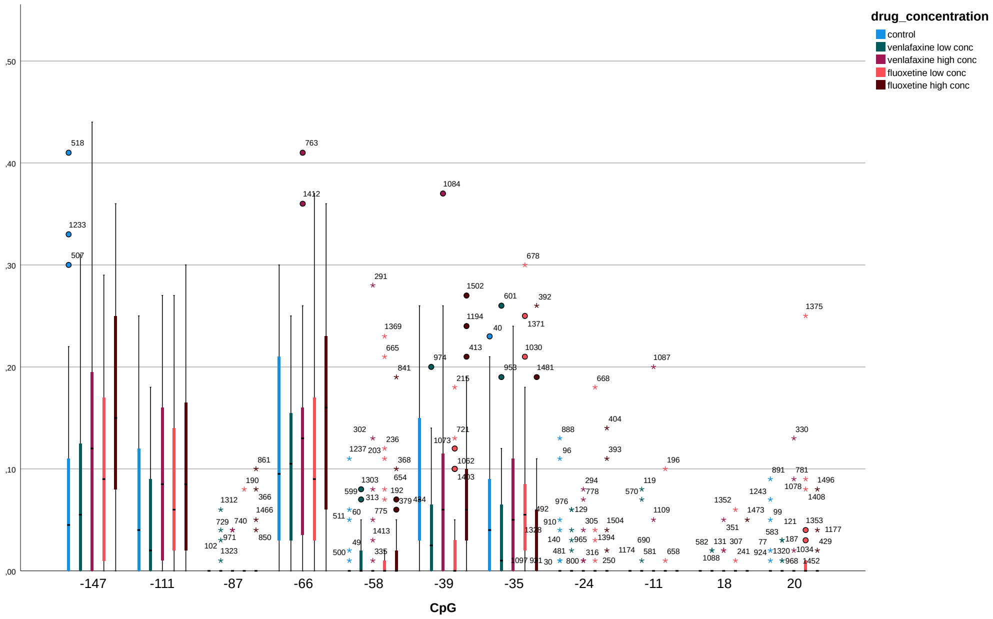

drug_concentration
control
venlafaxine low conc
venlafaxine high conc
fluoxetine low conc
fluoxetine high conc
,50
,40
,30
,20
,10
,00
-147
-111
-87
-66
-58
-39
-35
-24
-11
18
20
CpG
